# A System for the Qualitative Testing of Microfluidic Artificial Lungs Using Water

**DOI:** 10.7763/ijet.2024.v16.1277

**Published:** 2024

**Authors:** Kartik Tharwani, Gabriele K. Seilo, Jennifer Wang, Andrew Zhang, Joseph A. Potkay

**Affiliations:** 1 Department of Surgery, Joseph Potkay, University of Michigan, Ann Arbor, United States; 2 Ann Arbor Veterans Affairs Healthcare System, Joseph Potkay, University of Michigan, Ann Arbor, United States

**Keywords:** microfluidics, artificial lungs, gas exchange, qualitative testing, water-based testing

## Abstract

Microfluidic Artificial Lungs (μALs) are a promising technology for next generation artificial lungs, potentially offering improved treatment options for patients awaiting lung transplantation or requiring temporary respiratory support. Microfluidic artificial lungs are created using state-of-the-art manufacturing methods and can replicate the intricate flow networks of natural lungs and provide more efficient gas exchange. However, testing with blood is often labor intensive, logistically challenging, and expensive, which slows down the development of these technologies. We present a self-contained artificial lung testing system, addressing the challenges associated with traditional blood testing methods. To evaluate the artificial lungs’ gas exchange capabilities, water is employed as a safe, cheap, and convenient alternative to blood. pH measurements serve as a practical measure of carbon dioxide exchange, opposed to blood-based carbon dioxide measurements. Sensors are integrated into a single data logging system to reduce human error. The system successfully achieved qualitative gas exchange capabilities by adding and removing carbon dioxide from distilled water using 4 devices. Integrated pressure sensors measured pressure drop to determine fluidic resistance, providing insights into safe operational parameters. Finally, the system was demonstrated to be easily modified to evaluate oxygen exchange in blood, providing an easy transition to the next step of testing. The automated artificial lung testing system presents an alternative to conventional blood-based testing methods, offering cost-effective, safe, and efficient qualitative evaluation of microfluidic artificial lungs. This innovation streamlines μAL development, allowing for the faster development of this next generation artificial lung technology.

## I. Introduction

Artificial Lungs (ALs) act as a pivotal advancement in healthcare by providing a lifeline for patients grappling with End-Stage Lung Disease (ESLD) and Acute Respiratory Distress Syndrome (ARDS). Often caused by Chronic Obstructive Pulmonary Disease, ESLD affects approximately 5% of the US population [1]. Additionally, chronic lung disease is the fourth highest cause of death in the US [1]. ALs offer a treatment option for some of the challenges posed by organ transplantation, which often faces extensive waiting lists and stringent eligibility criteria. ARDS is a critical condition that can benefit from ALs. ARDS is characterized by rapid onset of severe respiratory failure, often resulting from factors such as severe infection, trauma, or inhalation of harmful substances. It leads to a significant decrease in lung function, making it challenging for patients to get enough oxygen [2]. By providing vital respiratory support, ALs can offer a temporary reprieve, allowing damaged lungs to heal or regain previous functionality (i.e., “bridge-to-recovery”). For chronic cases, ALs can serve as support until a transplant can be found. However, existing AL technology suffers from biocompatibility issues and exhibits a gas exchange efficiency far less than the natural lung. Addressing these shortcomings can potentially enhance quality of life and patient outcomes.

**Fig. 1.**
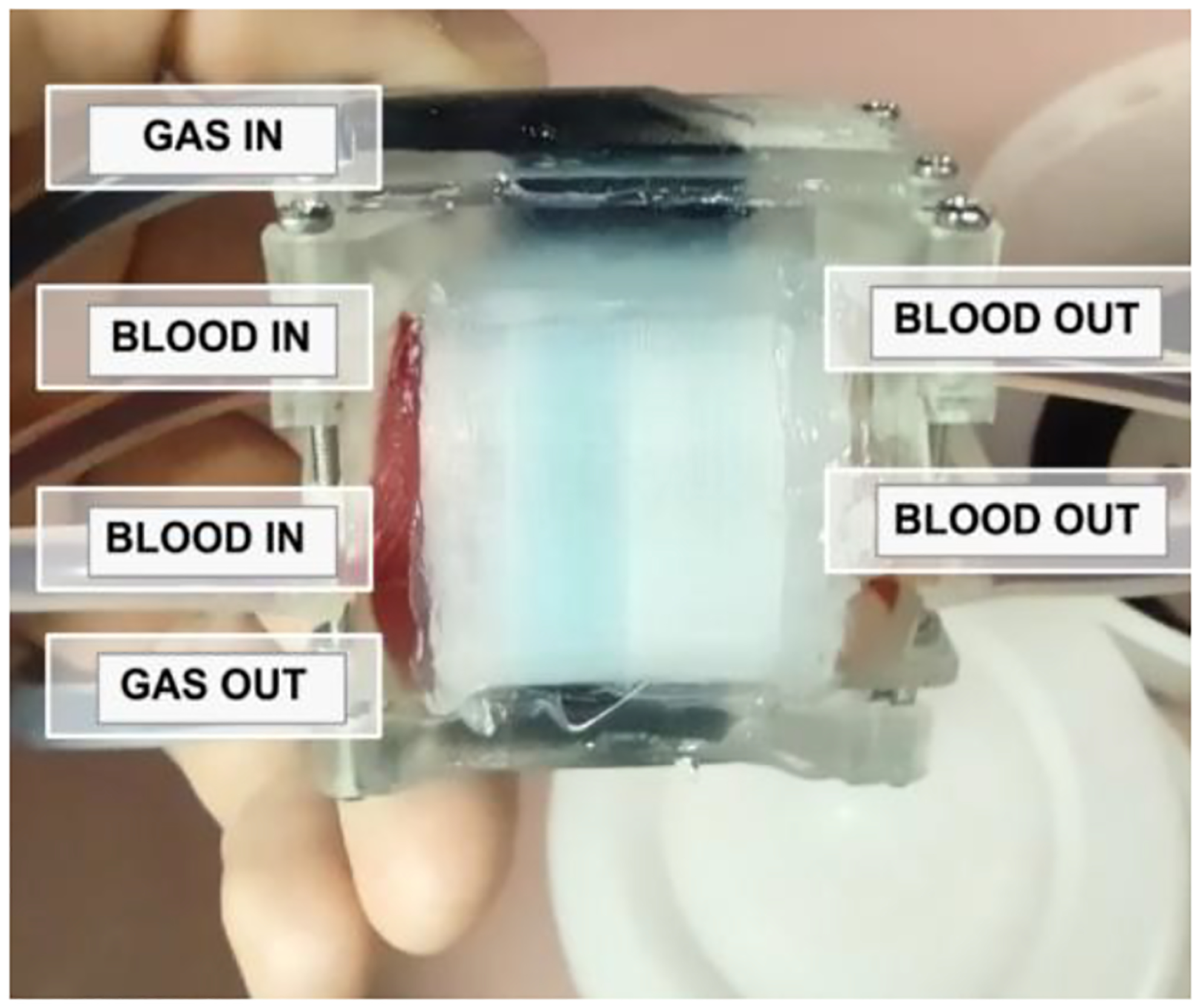
This lung (which will be introduced later as device C) was tested using the proposed system. Here, it is filled with food coloring for visualization. Testing is required to confirm this device can perform gas exchange due to the novel nature of its design.

Microfluidic Artificial Lungs (μALs) are an emerging technology holding remarkable promise in improving artificial lungs and the outcomes of patients on them. These devices better mimic the intricate microscale networks found in natural lungs, enabling more efficient gas exchange and reduced thrombosis risk (Fig. 1) [3]–[8].

**Fig. 2.**
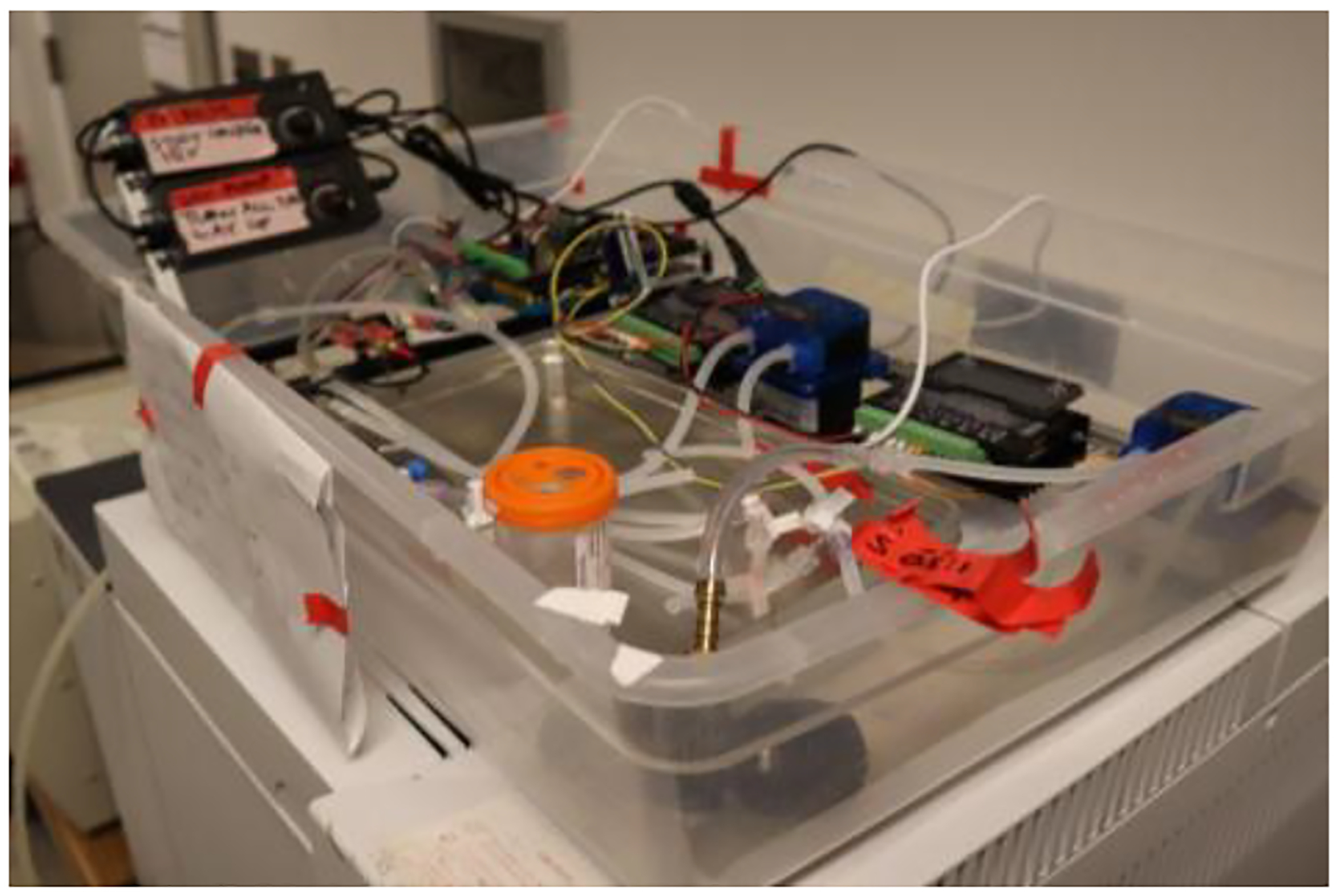
A self-contained, automated, μAL testing system would reduce many of the logistical challenges associated with testing μALs.

Recently, many new types of μALs have been developed with vastly varying manufacturing methods and characteristics. Typically, testing AL prototypes is a labor-intensive, expensive, and logistically complex process, requiring constant monitoring and adjustments [4, 7–14]. An automated, simplified testing method for testing the fluidic fidelity and functionality of ALs would speed up the development time of these devices (Fig. 2).

The necessity of blood-based testing brings forth a host of challenges that make the process exceedingly demanding. Blood is both a biohazard and a contamination risk. The requirement for specialized training limits the pool of individuals who can engage in testing procedures. Unlike water, blood cannot be sourced directly from a tap or easily stored due to issues such as bacterial infection and clotting. While the use of heparin can mitigate clotting, the time-sensitive nature and cost of using blood further complicate the testing process. Additionally, post-testing cleanup requires substantial effort. Finally, devices often cannot be fully cleaned, especially if there are clots, effectively making blood-testing a destructive assay method.

Given these challenges, water can serve as a cost-effective and safe alternative to avoid the logistical complexities associated with blood. Testing O_2_ exchange into water would not be representative of O_2_ exchange into blood because of the lack of hemoglobin. However, CO_2_ exchange between in-vitro water models has been shown to accurately reflect in-vivo studies [15].

Further, gas exchange via μALs relies on the interplay between gas and liquid flow networks. Gas and blood must be interwoven such that they are very close to each other (<100 μm) over a large surface area. However, there must not be a single leak from the gas to the blood side, or life-threatening gas embolisms will form. Finally, the membrane must exhibit permeability to both oxygen and CO_2_. This implies all material used in the fabrication of a μAL, which can facilitate CO_2_ exchange, fulfill these criteria, and ensure their capability for oxygen exchange as well. Therefore, though CO_2_ exchange cannot yield a quantitative value for O_2_ exchange, it can still be used as a qualitative indicator.

## II. System Design

### A. Rationale for Measuring pH

In this system, pH measurements are employed as a practical alternative to direct CO_2_ measurements in water. Proper storage, calibration, and handling of dissolved CO_2_ measurement probes involves significantly more complexity and cost compared to pH probes [16]. Directly measuring CO_2_ would eliminate many of the advantages hoped to be gained by this study. Measuring pH reduces both logistics and cost during testing.

### B. Blood Flow Network

**Fig. 3.**
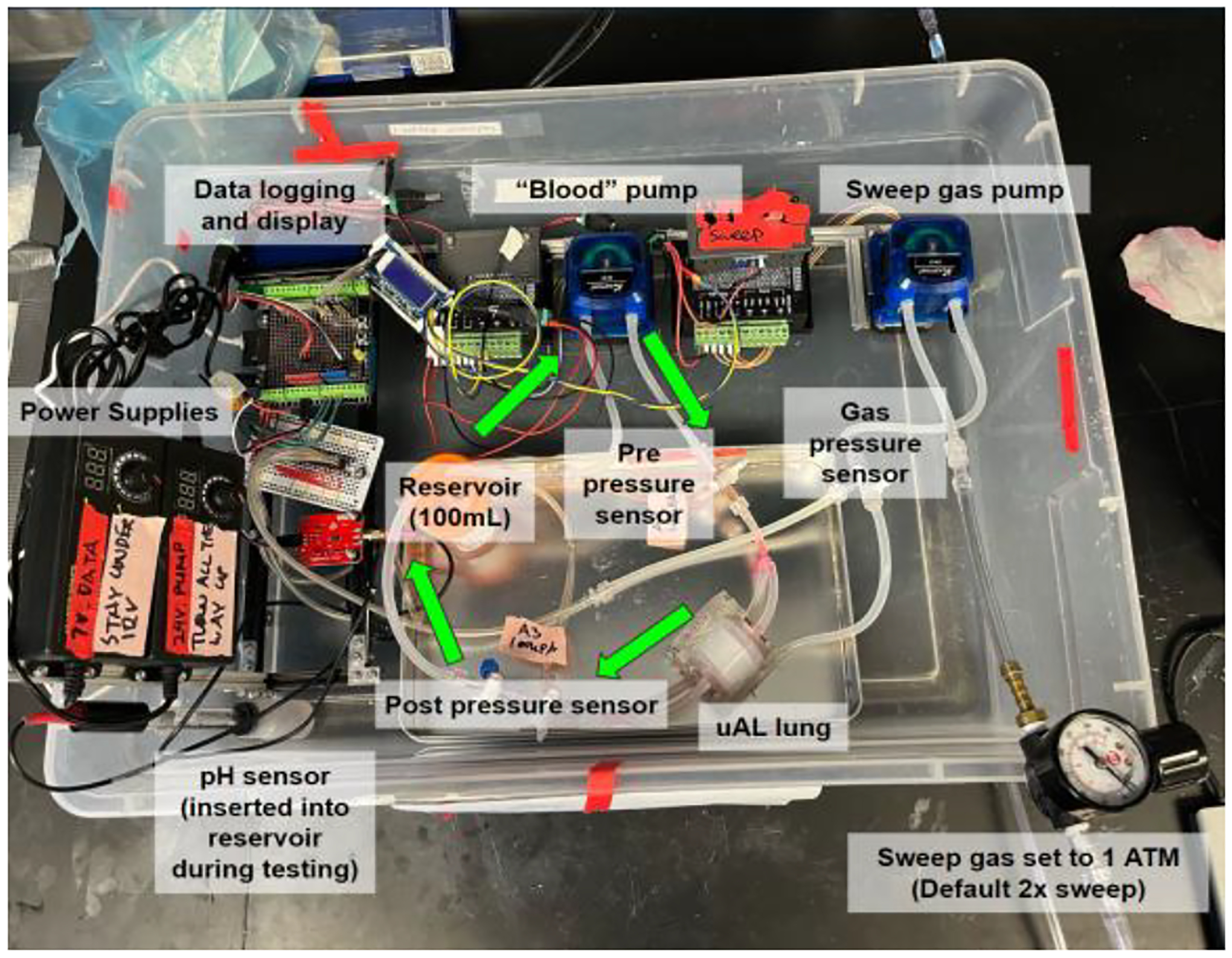
Complete system design for μAL testing.

The testing system encompasses two distinct flow networks: the “liquid” flow network (i.e., the blood or water flow circuit) and the “gas” flow network. They meet in the artificial lung, where gas exchange occurs (Fig. 3).

The liquid flow network starts with a reservoir containing approximately 100 mL of deionized water incorporating a pH sensor (Atlas Scientific Gravity pH Sensor). This reservoir serves as the initial source of the circulating fluid. A dual-channel peristaltic pump (Kamoer KPST-N14-C) takes water from the reservoir via flexible silicone tubing. The pump then pushes the fluid through the μAL’s liquid side. The pump has two channels, which can be put in parallel for high flow, in series for high pressure, or independently if the AL has two separate inlets requiring equal flow.

Within the μAL, the liquid flows through the channels within the lung structure, enabling gas exchange. Here, the “blood” exchanges CO_2_ with the gas, changing the pH accordingly. The pressure drop across the lung is monitored by two pressure sensors (Panasonic ADP5140) placed before and after the device. After the fluid progresses through the lung, it is channeled back to the reservoir, where its new pH is detected by the pH sensor. The cyclical configuration allows repeated and continuous simulations without needing to replace the water.

### C. Gas Flow Network

The gas flow network begins with a gas tank equipped with a regulator to modulate the gas pressure. A small peristaltic pump, placed in series with the regulator, ensures precise control over the gas flow rate into the system, see Fig. 3.

The chosen gasses for simulation, including compressed air (21% O_2_, 0% CO_2_), pure oxygen (100% O_2_), or pure carbon dioxide (100% CO_2_), are introduced into the μAL through the gas inlet. The gas pressure is set to 2 atmospheric pressures so that a fixed volume of gas released by the pump is equivalent to double that volume under standard conditions. This simplifies calculations. The pressure drop across the device is monitored by a single pressure sensor on the gas inlet. After passing through the device where gasses are exchanged with the liquid flow network, the gas is released into the atmosphere.

### D. Electrical Design

**Fig. 4.**
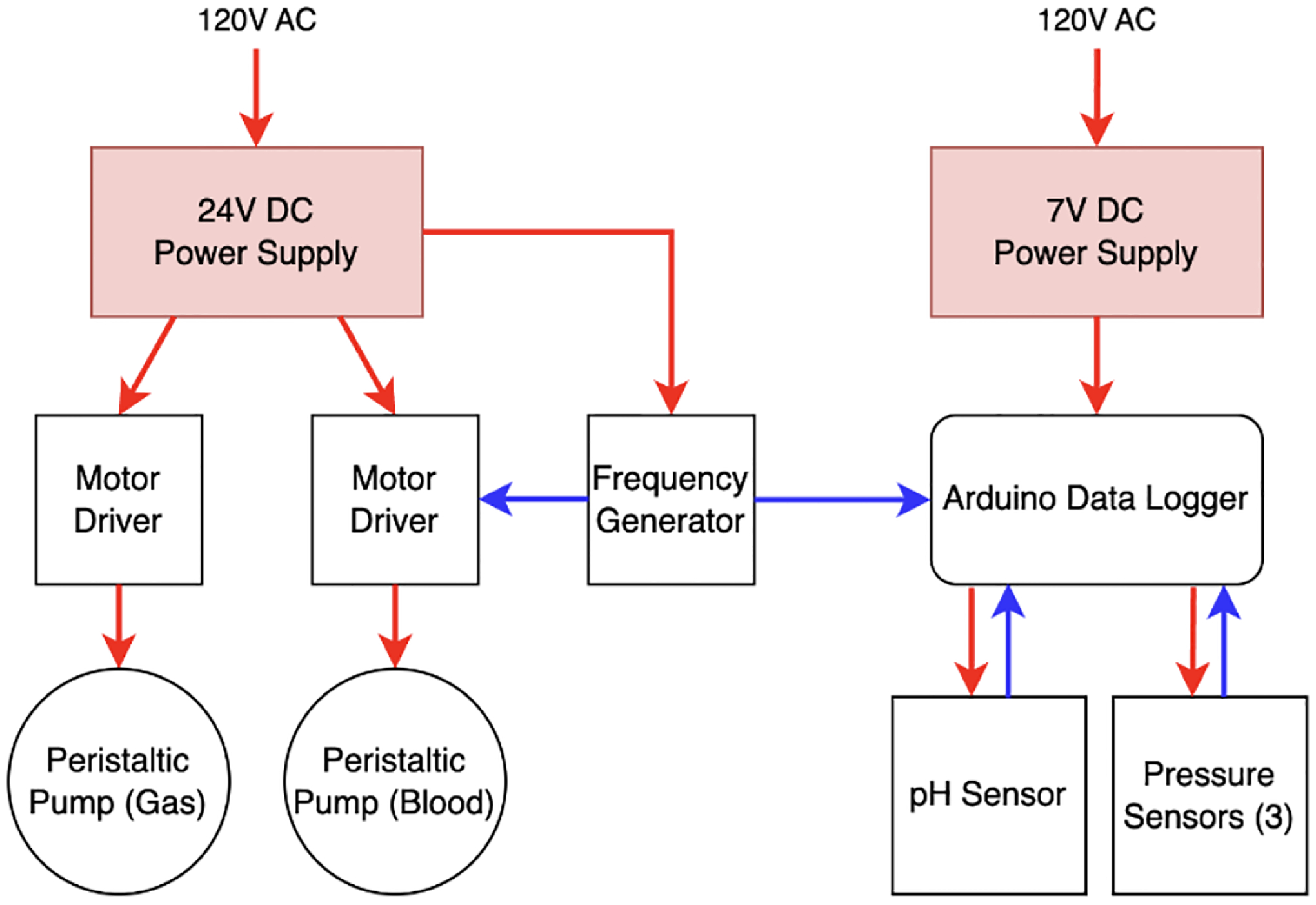
Electrical design for μAL testing setup (Red: power, blue: information).

The testing setup’s electrical architecture is divided into two distinct circuits: the pump circuit and the data circuit, offering independent control and measurement capabilities and enabling data logging to be performed even if the pumps are off, see Fig. 4.

The pump circuit powers the liquid pump and gas pump. Each pump is controlled by its own motor driver (TB6600). A frequency generator (XY-LPWM) controls the speed of both motor drivers. The drivers used in this part of the setup each have their own clock divider. This allows the blood and gas pumps to work at different ratios to each other, while the base speed is controlled by the frequency generator.

The data circuit focuses on acquiring, recording, and displaying vital information from the testing process. An Arduino microcontroller (Arduino Uno) is powered with a 7V power supply (LGY-002-2A). The Arduino collects data from both the pressure sensor and the pH sensor as well as the frequency generator.

The collected data, during testing, are channeled into a data logger (Adafruit Data Logging Shield). The Arduino orchestrates the creation of new data files on the SD card on the data logger, recording the readings from the sensors throughout the testing process. Simultaneously, an LCD display (LCD2004) allows real-time visualization of key metrics for the operator.

### E. Device under Test

The system was tested on a representative cylindrical μAL of unknown characteristics at the beginning of the study, See Fig. 1.

## III. Pressure and Flow Testing

### A. Purpose

Prior to testing gas exchange, the pressure drop of the μAL at various flow rates was evaluated. Pressure drop data were gathered by increasing the pump frequency (Hz) and thus pressure drop over time. This test determines gross functionality (lack of fluidic leaks), flow rate vs pressure drop (and fluidic resistance), and potentially the pressure at which a device might fail due to bursting, should failure occur. The pressure vs flow data eventually determine what clinical applications the μAL could operate in: pumped, systemic arterial-venous pumpless, or pulmonary arterial-venous pumpless.

### B. Method

Flow rate is increased logarithmically every two minutes by increments of approximately 10^1/6^ (10 Hz, 15 Hz, 22 Hz, 33 Hz, 47 Hz, 68 Hz, 100 Hz, and so-forth) [17]. The experiment is terminated upon reaching a predefined pressure threshold (200 mmHg for the devices under examination) or when the flow rate attains a predetermined target value (xx ml/min). At each flow rate, raw pressure data are averaged for 60 s for each reported pressure measurement. Filtering the data improves accuracy and accounts for the time it takes for the system to adjust to the increased flow rate.

The μAL under test had a particularly high resistance in the gas-flow network, so the gas: blood ratio was set to 0.5:1.0.

### C. Results and Analysis

**Fig. 5.**
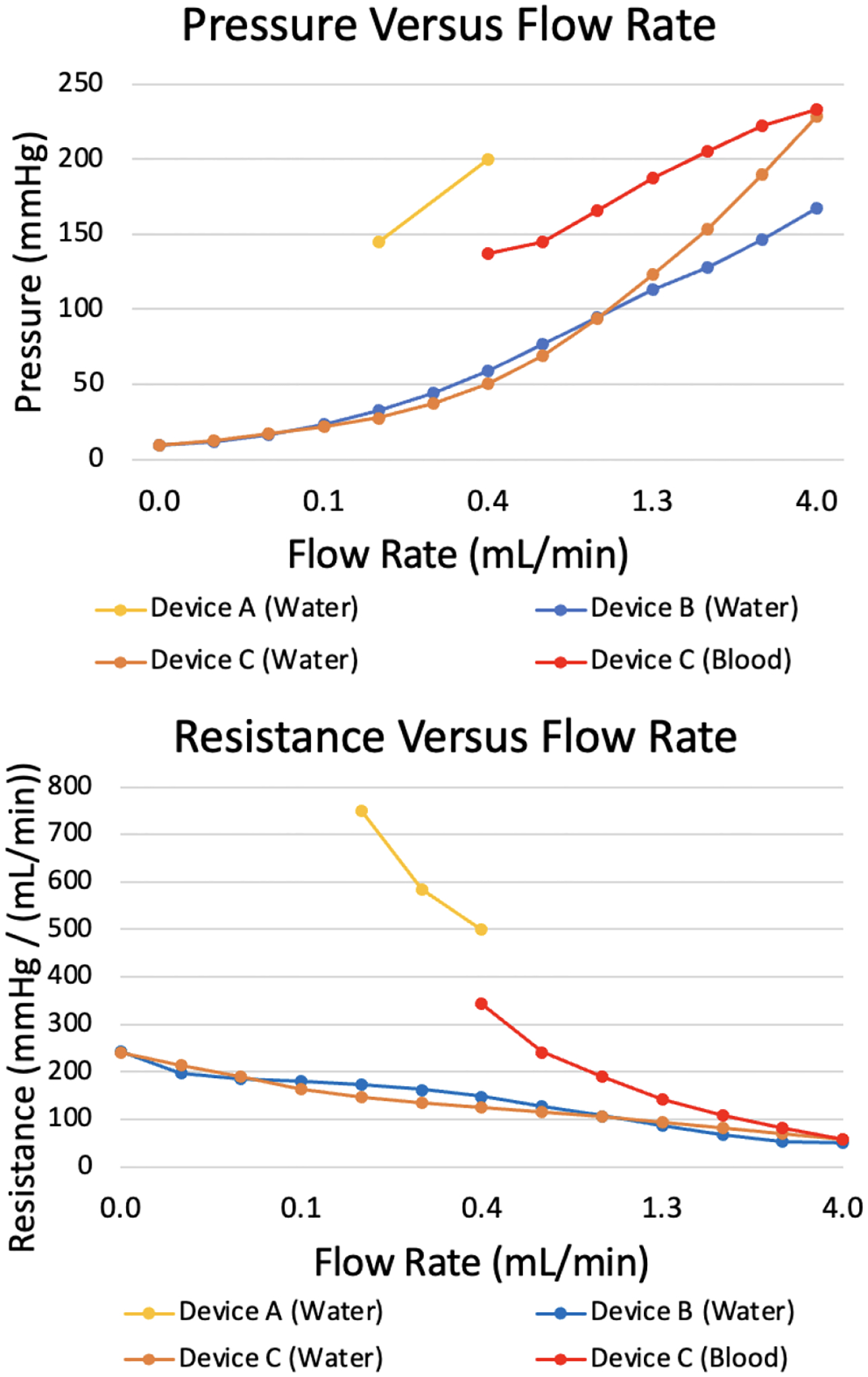
Pressure and resistance graphs detailing testing results for multiple completed μAL devices. Device B was also tested with blood, as will be covered under “Blood Testing”. The results of the blood test are also displayed here.

Pressure drop versus flow rate for four μAL devices is provided in Fig. 5 for flow rates up to 4 mL/min, demonstrating successful operation of the testing system. For each device, increasing flow rate correlates to increasing pressure, as expected. In this particular device design, the liquid side fluidic resistance decreases with increasing flow rate correlates to a lower resistance. Maximum flow rate was selected to be 4 mL/min. The pressure drop evaluation method is demonstrated to work.

## IV. CO_2_ Transfer Testing via Water pH

### A. Purpose

Water pH testing is performed to qualitatively evaluate the gas exchange functionality and capability of μALs. The primary objective of this test is to observe and measure pH changes within the system as the gas source is alternated between 0% and 100% CO_2_. The pH values obtained will provide insights into the effectiveness of our artificial lung in facilitating gas exchange.

### B. Method

To ensure accurate pH readings, a pre-calibration and post-calibration step is incorporated at the beginning and end of the test. Calibration is carried out using pH 7 and pH 4 buffer solutions. The pH sensors within our μAL testing system are calibrated against these known pH values, enhancing the reliability and accuracy of our pH measurements. Then, the water is conditioned by running the system with compressed gas over 24 hours to remove all CO_2_ dissolved in water. These calibrations and pre-conditioning could also be forgone, considering the qualitative nature of this test.

The gas exchange test involves switching the source gas in the μAL testing system from 0% to 100% CO_2_. When CO_2_ is exchanged in the μAL and dissolves into water on the liquid side of the μAL, it forms carbonic acid (H_2_CO_3_), which can further dissociate into bicarbonate ions (HCO_3_-) and hydrogen ions (H+). This process leads to a decrease in pH, causing the solution to become more acidic. After adding CO_2_ to the water, it is then removed by switching the source gas to 0% CO_2_, causing the pH to rise.

### C. Results and Analysis

**Fig. 6.**
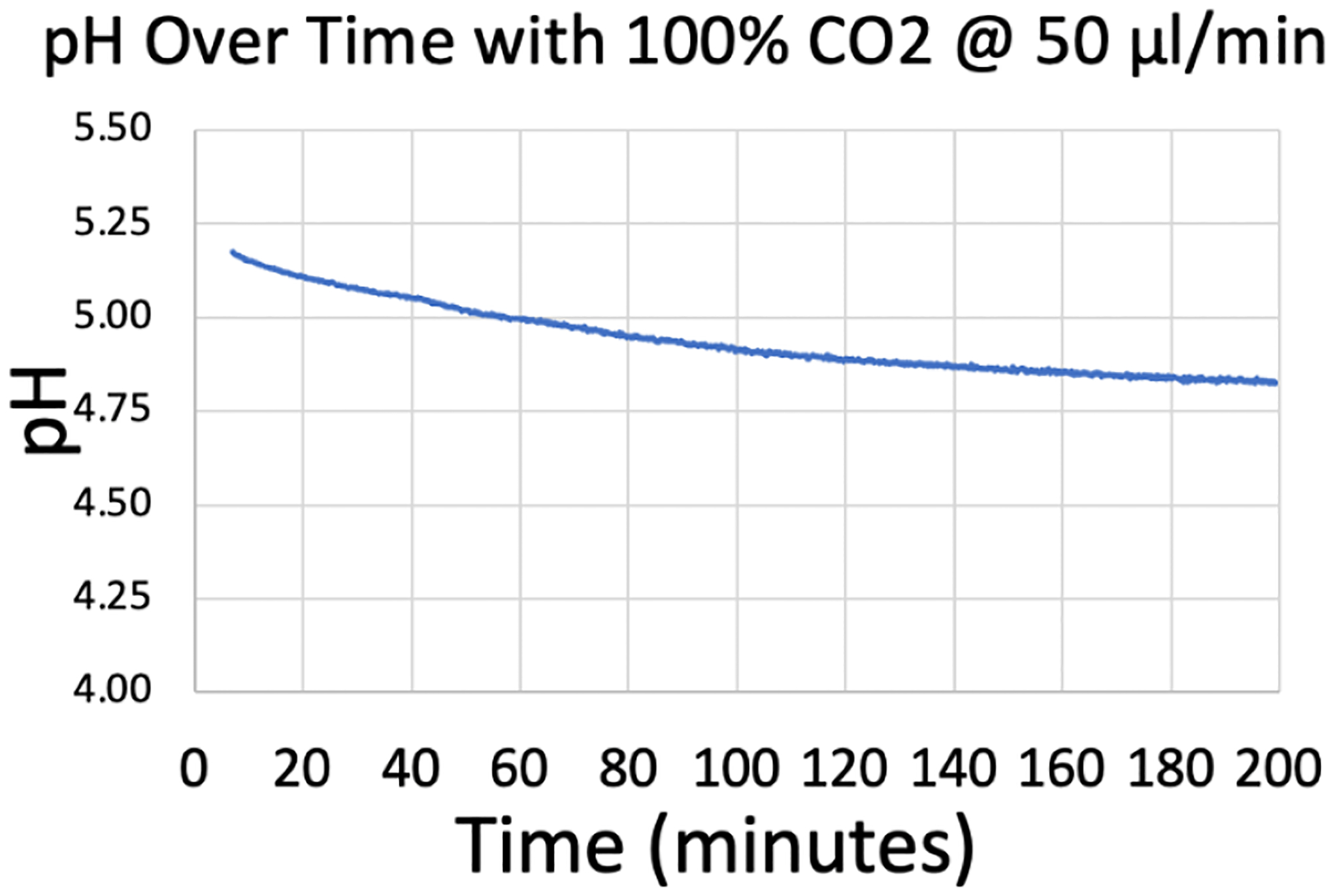
Device a demonstrated reduced the pH of water passing through the system when supplied with 100% CO_2_.

Device A underwent a partial test, without the pH calibration, without conditioning, and only adding CO_2_. Over ~3 hours, the device reduced the pH of the water from 5.2 to 4.8 (Fig. 6), demonstrating successful operation of the μAL testing system and the qualitative gas exchange capabilities of the μAL.

**Fig. 7.**
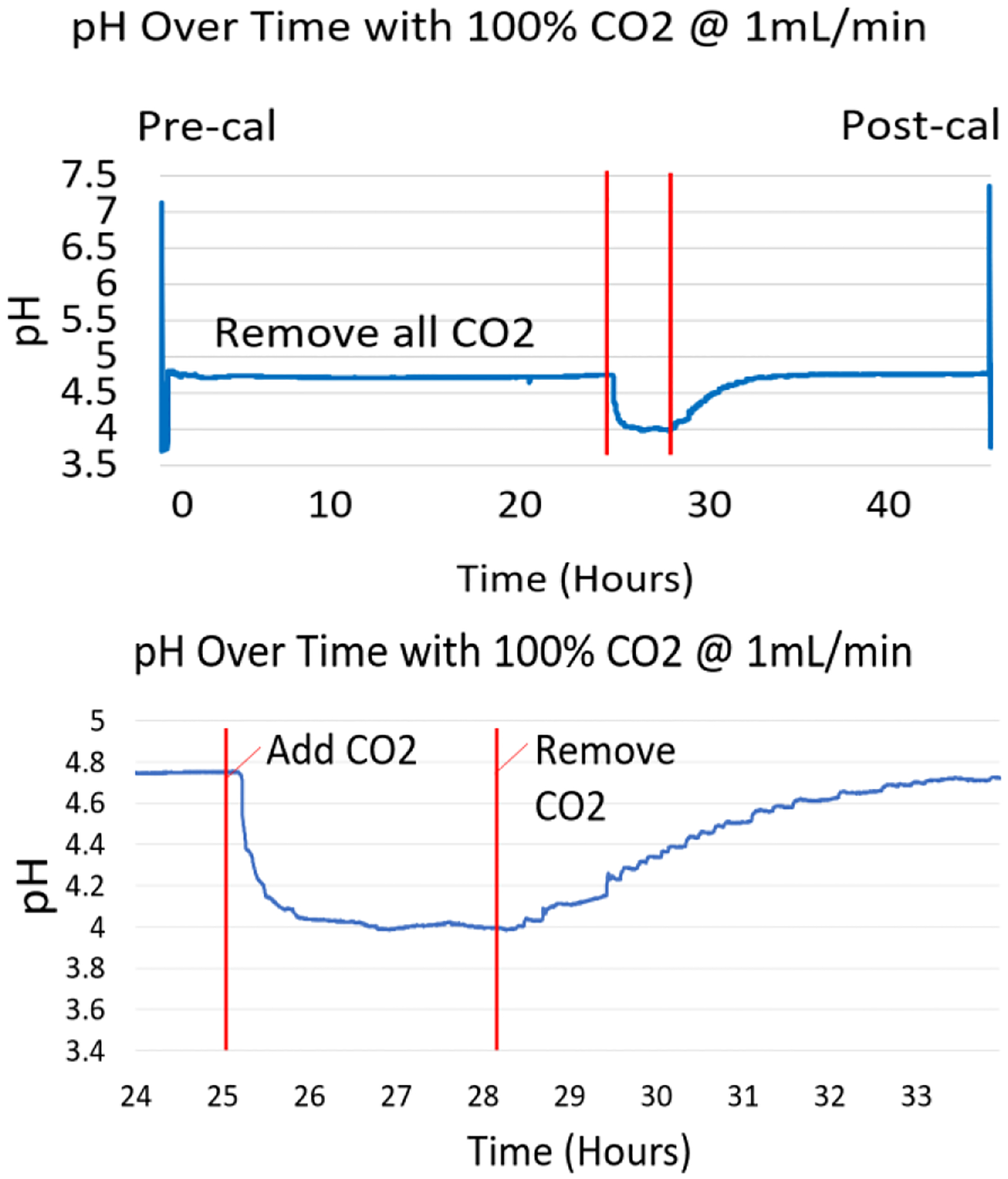
The full test with device B took 3 days (top). The pH shifts at Time=0h and Time=46h represent the pre and post calibration. The system is allowed to “settle” at 0% CO_2_ for a day. The area between the two red lines represents when the CO_2_ is set to 100% for 3 hours, and a corresponding drop and then rise in pH can be seen. In retrospect, the experiment could have been performed in 12 hours (bottom).

Device B underwent the entire pH testing process. The results in Fig. 7 show that when 100% CO_2_ gas flow is initiated, pH drops from approximately 4.75 to approximately 4.0 in 180 minutes.

Device C was damaged during setup and pH data were not collected.

## V. Blood Testing

### A. Purpose

In this experiment, the rated blood flow of Device B was determined following FDA guidance documents. Rated blood flow is the flow rate at which blood entering the μAL device at 70% O_2_ saturation would exit at 95% saturation, generally considered the primary metric of an AL’s performance. Pressure data is recorded to characterize pressure drop using blood instead of water. This evaluation aimed to achieve two objectives:

Firstly, it sought to substantiate the pH test methodology by confirming the devices capability to facilitate effective oxygen exchange, as initially indicated by the qualitative pH test.

Secondly, it demonstrates that the system can be easily modified to perform this rated-flow blood test. If a device is demonstrated to be capable of performing gas exchange based on the prior pH test with water, the next step is to perform an FDA rated-flow test using blood to quantify this capability. Therefore, a simple modification of the system to perform a blood test would save the trouble of setting up a separate testing setup for blood, moving the AL over, and setting up data logging and control systems for that separate system.

### B. Method

**Fig. 8.**
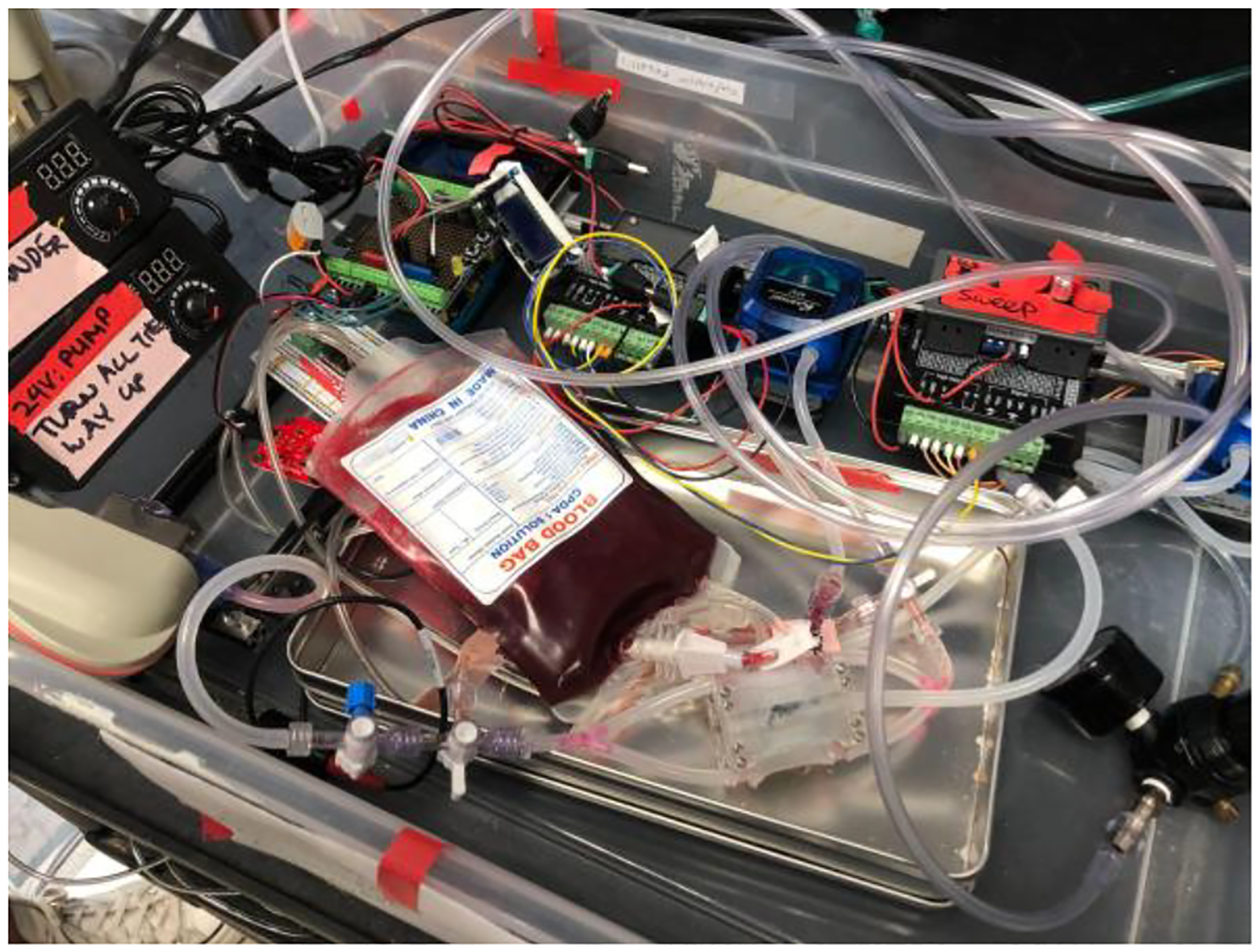
Blood testing setup after successful μAL water testing.

To assess the functional capability of the μAL using blood, a modified testing procedure was implemented (Fig. 8). In this modified setup, the reservoir was replaced with 250 mL of pre-conditioned blood, prepared in accordance with FDA regulations (65 ± 5% O_2_ saturation, hemoglobin concentration 12 ± 1 g/dL, pCO_2_ 45 ± 5 mmHg, base excess 0 ± 5 mmol/L, temperature: 37 ± 2°C, and pH:7.4 ± 0.1) A Terumo CDI-500 Sensor was placed after the μAL to monitor the processed blood, measuring pH, O_2_ partial pressure, O_2_ saturation, and CO_2_ partial pressure. After passing through the CDI-500 sensor, the blood was discarded into a waste bucket, as reintroducing it to the reservoir would cause the remaining blood in the reservoir to no longer meet FDA requirements. Gas flow was 100% O_2_ at a gas flow to blood flow ratio of 0.5:1.0.

Since the approximate safe operating parameters were already established with water, the test procedure can safely start at higher flows knowing that those flows would not damage the device. This greatly reduces the amount of testing that needs to be performed with blood.

### C. Results and Analysis

Pressure drop vs flow data are shown in Fig. 5. The resulting fluidic pressure drop with blood was approximately 70 mmHg greater than the resistance of Device B with water, consistent with the larger viscosity of blood compared to water.

**Fig. 9.**
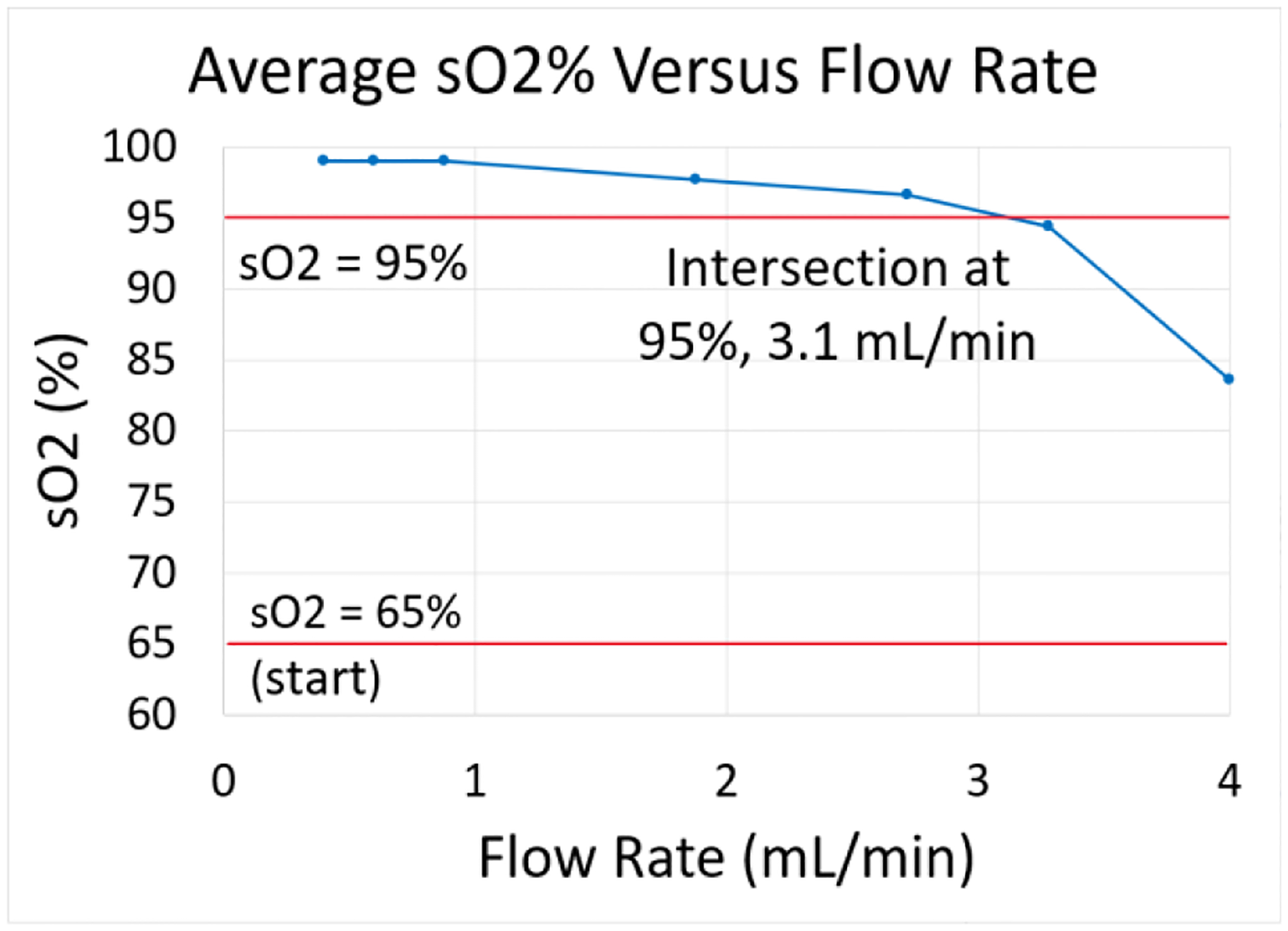
Evaluation of rated flow of the device. An extra data point is measured at 820 Hz to get a more accurate estimation of where O_2_ saturation (SO_2_) crossed 95%.

Outlet blood O_2_ readings were 100% at low flow rates and slowly decreased with increasing blood flow rate, as to be expected [18]. Measured outlet blood O_2_ saturation was 95% at a flow rate of approximately 3.1 mL/min, indicating the device’s rated blood flow, see Fig. 9. This test demonstrated that the device could indeed exchange O_2_, as suggested qualitatively by the pH test.

Blood testing started at 0.4 mL/min, compared to the 0.04 mL/min from water testing. This reduced the number of data points that needed to be gathered with blood by 6.

After the test, the μAL was removed, and hydrogen peroxide was pumped through the system to clean it. No residual blood was noticed throughout the testing system after the cleaning process, demonstrating that the testing system can continue to be used after a blood test.

## VI. Discussion

### A. Quantitatively Measuring Partial Pressure of CO_2_ and O_2_

Adding a water O_2_ sensor (significantly cheaper than a water CO_2_ sensor) was not done given that O_2_ storage and transport in water and blood are significantly different due to hemoglobin. Again, quantitative values for O_2_ were obtained during the blood testing, so this information would have been redundant.

Water CO_2_ was not quantitatively measured because the sensors were expensive and difficult to use. It would be theoretically possible to use pH to calculate partial pressure of CO_2_ in blood exchange. However, this would require the water to be highly pure. Ultimately, quantitative blood CO_2_ partial pressures were being provided by the CDI-500 sensor during the blood testing (although they were not recorded in this study), and so the additional effort to measure water CO_2_ partial pressures would have had limited benefit.

### B. Quantitatively CO_2_ and O_2_ Exchange

Total O_2_ and CO_2_ exchange can also be determined by comparing gas concentrations of O_2_ and CO_2_ between the gas inlet and outlet. This is typically more accurate because CO_2_ dissolved in water will become H_2_CO_3_, and so the partial pressure of CO_2_ is not directly translatable into dissolved CO_2_ [19]. Therefore, such gaseous sensors could be added to this system to further validate CO_2_ exchange performance. Dissolved O_2_ in blood can be determined through blood saturation measurements (with hemoglobin) but could also be validated by gaseous O_2_ measurements.

### C. Limits on Maximum Flow Rate

The pumps used in the system are limited to maximum flow rates of 60 mL/min. Researchers have demonstrated μALs with flow rates in the 100s of mL/min range [20]. To accommodate these devices, the pumps would need to be upgraded, potentially by combining two pumps: the current one covering the 1 mL/min to 50 mL/min range, and a second one from 50 mL/min to 500 mL/min. Above 500 mL/min, testing setups for clinical neonatal devices can be used.

### D. Potential Application in Coagulation Biocompatibility Studies

Although primarily intended as a system to determine the qualitative flow and gas exchange functionality of μAL, the presented system automatically measures pressure drop in blood, potentially enabling it to be used in blood coagulation studies. In these studies, device resistance (measured via pressure drop and flow readings) would serve as a measure of clotting within the μAL over time and thus of its blood compatibility. This system could thus be used as one measure to measure/compare the blood compatibility of different μAL designs.

## VII. Conclusion

This paper introduces a self-contained μAL testing system addressing the challenges associated with traditional testing methods, including complexity, cost, and safety. The system evaluates pressure drop data to establish safe operational parameters, confirms gas exchange without needing blood, and can be easily modified to perform blood testing when the time comes. Further, all sensors are integrated into a single data acquisition system to minimize required labor. This innovation enhances the speed, reliability, and accessibility of μAL development, paving the way to safer and more effective artificial lungs for patients requiring heart and lung support in the future.
